# Navigating the Complexity of Alternating Hemiplegia in Childhood: A Comprehensive Review

**DOI:** 10.5041/RMMJ.10529

**Published:** 2024-07-30

**Authors:** Jamir Pitton Rissardo, Nilofar Murtaza Vora, Yogendra Singh, Sweta Kishore, Ana Letícia Fornari Caprara

**Affiliations:** 1 Neurology Department, Cooper University Hospital, Camden, New Jersey, USA; 2 Medicine Department, Terna Speciality Hospital and Research Centre, Navi Mumbai, India; 3 Medicine Department, Federal University of Santa Maria, Santa Maria, Brazil

**Keywords:** Aicardi, alternating hemiplegia of childhood, *ATP1A2*, *ATP1A3*, flunarizine, rare disease

## Abstract

Alternating hemiplegia of childhood (AHC) is a complex neurodevelopmental disorder characterized by paroxysmal and transient events of unilateral or bilateral paresis, usually occurring before 18 months of age. Mutations in the *ATP1A3* gene, mainly p.Asp801Asn, p.Glu815Lys, and p.Gly947Arg at the protein level, are found in around 80% of the individuals with AHC. Interestingly, these mutations reflect the degree of severity of the neurological symptoms (p.Glu815Lys > p.Asp801Asn > p.Gly947Arg). Some channels involved in this disorder are N-type voltage-gated calcium channels, ATP-sensitive potassium channels, and the sodium/calcium exchanger. In this context, the management of AHC should be divided into the treatment of attacks, prophylactic treatment, and management of comorbidities commonly found in this group of individuals, including epilepsy, attention-deficit/hyperactivity disorder, aggressive behavior, cognitive impairment, movement disorders, and migraine. The importance of an integrated approach with a multidisciplinary team, such as neuropsychologists and dietitians, is worth mentioning, as well as the follow-up with a neurologist. In the present study, we propose new diagnostic criteria for AHC, dividing it into clinical, laboratory, supporting, and atypical features. Also, we review the location of the mutations in the *ATP1A3* protein of individuals with AHC, rapid-onset dystonia-parkinsonism (RDP) variants, and early infantile epileptic encephalopathy (variants with hemiplegic attack). We also include a section about the animal models for *ATP1A3* disorders.

## INTRODUCTION

Alternating hemiplegia of childhood (AHC) is a complex neurodevelopmental disorder mainly characterized by paroxysmal transient events of unilateral or bilateral paresis, usually before 18 months.^1^ It is a rare and severe neurological disorder with a prevalence of 1:100,000 to 1:1,000,000.^2^ Little is known about the several genetic, environmental, and biological factors that could potentially contribute to the pathophysiology of AHC. In this way, diagnosis and management are empirical in many cases, with few evidence-based studies to support specific tests or therapeutic strategies.

The AHC disorder has been associated with mutations in the *ATP1A3* gene, which encodes the alpha-3 subunit of the neuronal Na^+^/K^+^-ATPase transmembrane ion pump in approximately 75% of patients.^3^ The *ATP1A3* gene is highly expressed in brain regions that influence the autonomic nervous system.^4^ In approximately a quarter of the patients, ATP1A2 mutations were encountered, while in a minority of the subjects, the etiology is still unknown.

The presentation of AHC is often associated with precipitating factors such as environmental and psychological stressors. Paroxysmal episodes may occur independently or in association with other clinical manifestations, such as autonomic dysfunction, altered mental status, and abnormal movements, such as dystonia, ataxia, and choreoathetosis.^5^

A typical clinical pattern can be observed in most individuals with AHC. In this way, it is possible to divide the clinical progression of the disease into three phases. The first phase occurs during the first 3 months of age, and the main clinical feature is abnormal ocular movements, which can be associated with dystonia.^1^ The second phase generally starts at 4 months and usually extends until 6 years; it is characterized by hemiplegic spells. In this phase, the patient can also present delayed developmental milestones and seizures.^5^ In the last phase, unremitting neurological deficits could be observed. Other significant findings in the last phase include persistent developmental delays and, less frequently, hemiplegic and dystonic spells.^6^

Patients with AHC have a significant burden of neurological comorbidities and frequently also have deficits in sustained attention, reduced speed of information processing, and difficulties in understanding, speaking, and with working memory.^7^ Moreover, patients often have gross motor function impairments, which can be complicated by attention-deficit/hyperactivity disorder, disruptive behavior, and anxiety disorders.^8^ Interestingly, some clinical manifestations occur in distinctive and sequential phases, disappearing during the sleep cycle.^9^

Advanced molecular research has allowed a better understanding of the main genes involved in this condition and contributes to early confirmation of the diagnosis. In this way, the present study aims to review the recent advancements in understanding the clinical features and pathophysiology of AHC, as well as current recommendations for genetic testing and treatment strategies.

## CLINICAL MANIFESTATIONS

### Onset and Frequency

Usually, AHC episodes start before 18 months of age and are generally related to specific triggers. The most common early clinical signs include autonomic dysfunctions associated with precipitating factors, ocular abnormalities, and tonic or dystonic episodes. Paroxysmal tonic or dystonic episodes frequently occur in one limb or, less frequently, a hemibody. The most common type of ocular abnormality reported is nystagmus, which usually affects only one eye and can manifest alone or in conjunction with dystonic or tonic symptoms.^10^

### Hemiplegia

Hemiplegia is the central and most prominent symptom of AHC. It involves a temporary loss of motor function on one side of the body during an episode. The paralysis can be partial or complete and is reversible, typically resolving once the episode subsides.^1^ The alternating pattern of hemiplegia is a distinguishing characteristic of AHC. This alternating paralysis can affect various body parts, including the face, arms, and legs. The hemiplegic episodes in their classical presentations are mild and irregular when they first start, but they eventually evolve into a characteristic clinical appearance.^8^ Plegic attacks can affect one side or, less often, both sides and can begin suddenly or develop gradually over several minutes. Episodes can last from a few minutes to several days and occur at varying frequencies.^9^ During an attack, the patient often maintains consciousness but is restless and anxious. Usually, the lower limbs are not as severely affected as the arms. Hemiplegia can alternate from side to side in the same episode.^5^

### Motor Symptoms

During hemiplegic episodes, there is a significant impairment of motor function on the affected side. This can manifest as weakness, stiffness, and difficulty in voluntary movements. Motor milestone delays are commonly reported. Bourgeois noted that 24 participants had poor fine-motor control and had only learned to walk independently at the average age of 44 months. Often, the first gross motor abnormality to be reported is hypotonia.^11^ Another study by Masoud et al. examined the functions of gross motor, upper extremity motor control, motor speech, and dysphagia in 23 AHC patients; 9 were males, and 14 were females, with a mean age of 9.33 years.^12^ Their findings indicated that speech impairments related to motor function were markedly more severe than gross motor abnormalities as determined by the Gross Motor Function Classification System (GMFCS). Only three patients were defined as moderate to severe according to the GMFCS criteria. Also, Masoud et al. recognized that the oropharyngeal function is one of the most severely impaired functional domains in patients with AHC.^12^

### Oculomotor Disturbances

The AHC disorder is associated with distinct oculomotor disturbances, encompassing several eye movement abnormalities. Nystagmus is frequently observed, but strabismus may also be present. These eye abnormalities can be exacerbated during episodes of hemiplegia, contributing to the complexity of the clinical presentation. Aicardi et al. reported a cohort of 20 patients, in which nystagmus was observed in 90%. The nystagmus was characterized as monocular in 14, horizontal in 13, and vertical in 3 patients.^10^ The nystagmus and hemiplegia typically alternate from eye to eye with each subsequent episode.^10^,^13^ Anisocoria, or a larger pupil contralateral to the abducting eye, was rarely described.^14^ During the spells, the non-abducting eye has been reported to have reduced horizontal but intact vertical movements.^15^ Interestingly, some patients can present horizontal abduction jerks of one eye. Still, the abnormal movements of the limbs can be accompanied by a tonic abducting deviation of the eye located on the contralateral side. When vertical nystagmus was observed, it affected both eyes with similar amplitudes.^16^

### Seizures

Seizures occur in a variety of forms, affecting a significant percentage of people with AHC. The most common seizure types in this population are atonic, focal, or generalized tonic–clonic seizures. In AHC, seizures can occur separately or with hemiplegic episodes. For these patients, seizures can be a major source of distress and stigmatization, contributing to social isolation and decreased quality of life, and also leading to an increased number of plegic attacks. Sweney et al. reported generalized, tonic, or tonic–clonic seizures in 44 out of 103 (43%) AHC patients, with the mean age of epileptic onset attacks being around the age of 6 years.^5^ Ten children (23%) did not have another seizure until they were 10 years old or older.^5^

### Cognitive and Developmental Challenges

Cognitive impairments are often seen in AHC, including learning difficulties, intellectual disability, and developmental delays. Additionally, Mikaki et al. observed delayed developmental milestones in 40 out of 44 (90%) of the patients with AHC. Also, they noted a correlation between cognitive impairment and the age of hemiplegic attack onset in these individuals.^1^ Therefore, the findings reported by Mikaki et al. raised questions about whether the cognitive impairment seen in people with AHC is a consequence of the neurological condition or a symptomatic manifestation of the paroxysmal episodes.^1^

### Autonomic Dysfunction

Some individuals with AHC experience autonomic dysfunction, which can manifest as problems in regulating heart rate, gastric emptying, and body temperature. The hypothalamus and vagus nerve nuclei, which regulate the autonomic nervous system, have high expression levels of *ATP1A3*.^4^,^17^ Additionally, this gene is expressed in gamma-aminobutyric acid interneurons that can regulate motility and in the motor brain stem nuclei that regulate swallowing.^18^

### Sleep Disturbances

An adequate sleep cycle has been shown to have a significant clinical benefit in managing plegic attacks, and sleep dysfunction has been observed in patients with AHC.^10^,^11^ Individuals with AHC often exhibit irregular sleep patterns. The alternating hemiplegic episodes, which characterize the condition, can disrupt the normal sleep–wake cycle, leading to inconsistencies in sleep duration and quality noticed in polysomnography studies. Dysautonomia, a common feature of AHC, can affect the autonomic control of respiration. Irregular respiratory patterns during sleep may impact sleep quality and potentially lead to sleep-related breathing disorders. A study by Kansagra et al. found that 20 of 22 patients with AHC had at least one type of sleep problem, of which 6 had obstructive sleep apnea syndrome, a high mean overall apnea-hypopnea score, and a high mean arousal index.^6^

### Neuropsychological Disturbances

Jasien et al. evaluated neuropsychological abnormalities in 25 individuals with AHC and found significant impairments in cognition, expressive and receptive language, executive function, attention, and behavior.^19^ In this study, 10 patients with AHC showed attention-deficit/hyperactivity disorder, 7 had disruptive behavior, and 3 had anxiety disorder.^19^ Eight out of 25 subjects exhibited impairments in comprehension and long-term memory, inadequate academic performance, and dyscalculia.^19^ In the same study, cognitive function was normal in 4 (16%), borderline in 3 (12%), and impaired in 18 (72%), of which 6 (24%) were mild, 10 (40%) moderate, and 2 (8%) severe.^19^

### Migraine

Migraine is not commonly reported in patients with AHC, and the prevalence varies widely in the literature. Sakuragawa reported that only 9.1% of the individuals had a family history of migraine, and 13% of the subjects reported headaches as a prodrome of the plegic attacks.^9^ Mikati et al. revealed a prevalence of 25% of migraine in immediate family members of individuals with AHC.^1^ Saito et al. reported a single case of migraine out of 10 in patients with AHC.^20^

## ETIOLOGY AND PATHOPHYSIOLOGY

### Genetic Basis

**Figure 1:**
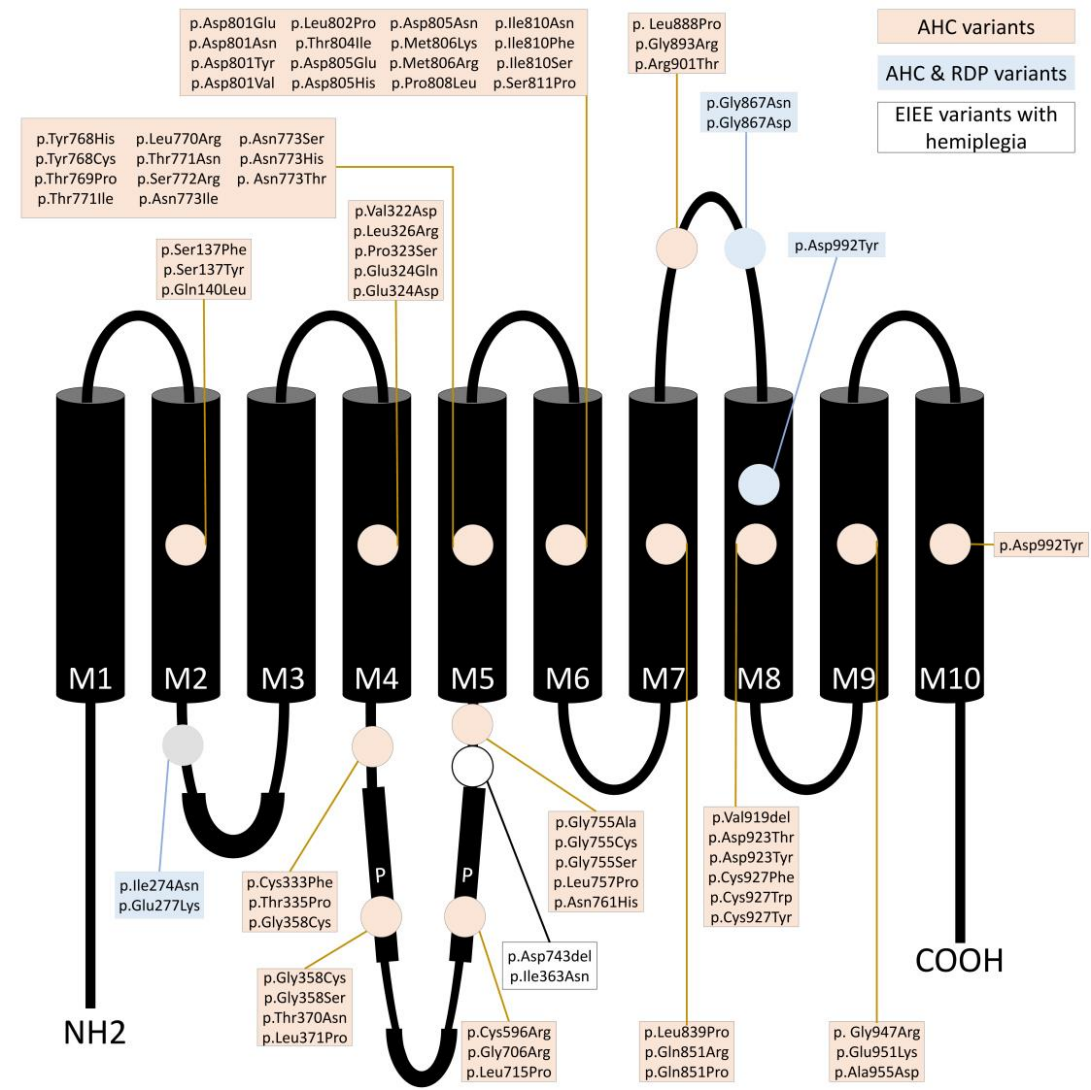
Location of Alternating Hemiplegia of Childhood (AHC) Variants in the *ATP1A3* Protein, Including Rapid-onset Dystonia-parkinsonism (RDP) Variants and Early Infantile Epileptic Encephalopathy (EIEE) Variants with Hemiplegic Attack. Light orange = AHC variants; light blue = AHC and RDP variants; white = EIEE variants with hemiplegia. Diagram based on Viollet et al. (2015),^23^ Roubergue et al. (2013),^24^ Boelman et al. (2014),^25^ and Kanavakis et al. (2003).^26^

Alternating hemiplegia of childhood is primarily associated with mutations in the *ATP1A3* gene on chromosome 19q13.^21^ This gene encodes the alpha-3 subunit of the Na^+^/K^+^-ATPase pump.^22^ Although AHC is a sporadic disorder caused by *de novo* variants, a few autosomal dominant inherited cases have been reported (Figure 1).^23^–^26^ Germline mosaicism has been described in familial cases of other *ATP1A3*-related disorders but has still not been reported in AHC.^27^

Some of the *ATP1A3*-related disorders are AHC, rapid-onset dystonia-parkinsonism (RDP), and cerebellar ataxia, areflexia, pes cavus, optic atrophy, and sensorineural hearing loss (CAPOS) syndrome.

Clinical data from a cohort of 155 patients from the International Consortium of AHC were carefully examined, and a distinct genotype-phenotype association study was performed.^28^ Interestingly, 85% of patients had *ATP1A3* mutations, indicating that a small subset fulfilled AHC criteria without any molecular genetic diagnosis. The study also confirmed the incidence of the three most common mutations: p.Asp801Asn, p.Glu815Lys, and p.Gly947Arg. Of these, p.Glu815Lys was associated with a more severe phenotype, with drug-resistant epilepsy, profound intellectual disability, and severe dystonia. The p.Asp801Asn mutation appears to be associated with a milder phenotype with later onset of the paroxysmal events and less frequent plegic attacks; the majority of these patients present moderate intellectual disability with a higher rate of behavioral problems. The presence of p.Gly947Arg correlates with a favorable prognosis. Also, paroxysmal events have a later onset with p.Gly947Arg compared to the other two mutations. Moreover, no severe intellectual disability has been reported with p.Gly947Arg. In conclusion, the three mutations show a gradient of the severity of the symptoms: p.Glu815Lys > p.Asp801Asn > p.Gly947Arg.^28^

Table 1 lists the percentages of the three most frequent mutations encountered in individuals with AHC in different cohorts (Table 1).^3^,^23^,^28^–^36^ Prognosis can be reasonably predicted for patients with these recurrent *ATP1A3* mutations. However, more cohort studies are needed in the literature for a complete understanding of the best therapeutic choices and clinical course of this rare syndrome. Also, the present cohorts in the literature provide symptoms not commonly seen in individuals with AHC but usually seen in other *ATP1A3* disorders. This overlap of clinical manifestations may suggest a continuum spectrum of the disorders related to this genetic mutation.

**Table 1:** Frequency of Alternating Hemiplegia of Childhood Variants in Different Cohorts.

Reference	Population	*n*	Percentage of Mutations			
			*p.Asp801Asn*	*p.Glu815Lys*	*p.Gly947Arg*	Others
Cordani et al. (2021)^29^	European, Italy	39	26%	23%	10%	41%
Gurrieri et al. (2016)^30^	European, Italy	30	37%	37%	20%	6%
Viollet et al. (2015)^23^	North American	154	40%	26%	8%	26%
Panagiotakaki et al. (2015)^28^	European	155	43%	16%	11%	30%
Sasaki et al. (2014)^31^	Japanese	35	30%	36%	3%	31%
Yang et al. (2014)^32^	Chinese	47	31%	20%	15%	34%
Hoei-Hansen et al. (2014)^3^	European, Denmark	10	20%	10%	20%	50%
Vila-Pueyo et al. (2014)^33^	European	10	10%	10%	30%	50%
Ishii et al. (2013)^34^	Japanese	8	37%	37%	NA	26%
Rosewich et al. (2012)^35^	European, Germany	15	38%	29%	NA	33%
Heinzen et al. (2012)^36^	Australian, European, and North American	98	34%	18%	7%	37%

*n*, number of participants in the study; NA, not available/not applicable.

### Ion Transport Dysfunction

**Figure 2:**
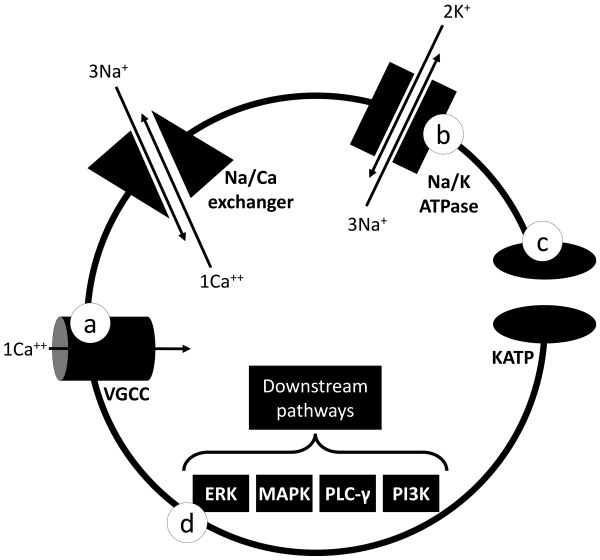
Schematic Representation of the Downstream Pathways and the Na^+^-K^+^-ATPase at the Cell Membrane. Also, the mechanism of some therapies related to the management of alternating hemiplegia of childhood is represented: flunarizine (**a**), rostafuroxin (**b**), ketogenic diet (**c**), and SL327, which inhibits ERK-kinase (MEK)-1 and MEK2 (**d**). Ca, calcium; ERK, extracellular signal-regulated kinases; K, potassium; KATP, ATP-sensitive potassium channel; MAPK, mitogen-activated protein kinases; Na, sodium; PI3K, phosphoinositide 3-kinases; PLC-γ, phospholipase C gamma; VGCC, voltage-gated calcium channels of N-type.

The Na^+^/K^+^-ATPase pump is fundamental for maintaining the resting membrane potential of neurons (Figure 2). Disruption of this pump contributes to the hyperexcitability of neurons, abnormal firing patterns, and impaired regulation of neurotransmitter release. The mutations in *ATP1A3* result in dysfunctional sodium-potassium pump activity, leading to impaired ion transport and disrupted neuronal excitability. The alpha-3 subunit is present on the cytosolic side of the cell membrane of neurons and glial cells, which explains the neurological findings seen in AHC.^18^,^37^ In the neuron with the alpha-3 allele mutation, Na^+^/K^+^-ATPase has a reduced sodium affinity, resulting in an increased intracellular Na^+^ concentration and a variety of dramatic events such as increased calcium influx into the cell with lethal effects and the liberation of excitatory amino acids.^38^

Different pathogenic causes could explain heterogeneity in the phenotypic range of patients with AHC and RDP. Therefore, based on the description and characterization of *ATP1A3* mutations related to AHC, the activity of the Na^+^/K^+^-ATPase pump appears to be essential for pathogenicity in AHC.^36^ As a result, most RDP mutations likely influence protein expression and cell surface expression, but in AHC a change in the pump activity could explain the phenotype.

### Environmental and Epigenetic Modifiers

The most significant players in the pathophysiology of AHC are probably mutations related to *ATP1A3*. Still, environmental factors are believed to trigger or exacerbate episodes in genetically susceptible individuals. Physiological or psychological stressors usually trigger the onset of AHC symptoms.

Some triggers already reported in the literature are strenuous exercise, illness, prolonged fasting, and psychological stress. Also, environmental stressors (bright light, excessive heat or cold, noise, crowds), water exposure (bathing and swimming), and certain foods or odors (chocolate and food dyes) are associated with the occurrence of AHC symptoms.^39^ In this context, the interaction between genes and environmental factors may be responsible for the diverse phenotypes and disease spectrum.

## DIAGNOSIS AND DIFFERENTIAL DIAGNOSIS

Alternating hemiplegia of childhood is a rare disorder first reported in the medical literature in 1971 by Simon Verret and John C. Steele.^40^ Nevertheless, only two decades later, diagnostic criteria were proposed by Ingeborg Krägeloh-Mann and Jean Aicardi, who also emphasized that AHC was a nosologic entity distinct from hemiplegic migraine.^10^ There is no diagnostic test for AHC, and it remains a clinical diagnosis. The diagnosis can be obtained according to six clinical criteria, known as the Aicardi criteria.^10^ One of the cardinal diagnostic criteria of AHC is the remission of hemiplegia and other paroxysmal events, but not seizures, with sleep and their potential recurrence shortly after waking.^41^ However, in 2012, the molecular abnormality of *ATP1A3* was identified as the primary etiology of AHC (Table 2).^5^,^7^,^23^,^42^,^43^

**Table 2:** Diagnostic Criteria for Alternating Hemiplegia of Childhood Proposed by Rissardo et al.

Type of Criteria	Recommendations
Clinical criteria (Aicardi criteria)	Onset before 18 months of age Episodes of hemiplegia involving alternating body sides Bilateral hemiplegia or quadriplegia as a separate attack or as a generalization of a hemiplegic event Other paroxysmal movements (dystonic spells, abnormal oculomotor symptoms, or autonomic phenomenon) either concurrent with hemiplegic attacks or independently Disappearance of symptoms while sleeping, although attacks may resume soon after awakening Developmental delay or permanent neurological findings such as dystonia, ataxia, and choreoathetosis
Laboratory criterion	*ATP1A3* mutations, mainly at the p.Asp801Asn, p.Glu815Lys, or p.Gly947Arg protein level
Supporting criteria	Recurrent attacks of monocular nystagmus Neuroimaging within normal limits, despite the presence of permanent neurological findings Electroencephalography without abnormal electrical activity Stepwise milestone deterioration in the setting of paroxysmal attacks New permanent neurological deficit with a rostrocaudal gradient distribution Family history negative for hemiplegic symptoms or presence of dominant inheritance
Atypical features	Onset after 18 months Neuroimaging showing structural abnormalities at presentation

Diagnostic criteria are based on Rosewich et al. (2017),^42^ Viollet et al. (2015),^23^ Panagiotakaki et al. (2010),^43^ Sweney et al. (2009),^5^ and Bourgeois et al. (1993).^7^

Moyamoya angiopathy and mitochondrial diseases, such as Kearns–Sayre syndrome, mitochondrial encephalomyopathy, lactic acidosis, and stroke-like episodes, are a few disorders with clinical manifestations that may overlap with AHC. Moyamoya angiopathy presents with transient ischemic attack and visual disturbances, aphasia, weakness, numbness, or paralysis involving the face, arm, or leg, typically in one side of the body, mimicking the AHC episodes.^44^ It is a vascular disorder characterized by progressive stenosis of the terminal portion of the internal carotid arteries and the development of a network of abnormal collateral vessels in which the carotid artery becomes partially or completely occluded, compromising cerebral blood flow. Neuroimaging can help differentiate Moyamoya syndrome from AHC since Moyamoya syndrome could lead to strokes.

The AHC disorder may mimic Kearns–Sayre syndrome, a subtype of chronic progressive external ophthalmoplegia (CPEO). In addition to CPEO symptoms, Kearns–Sayre syndrome usually presents before the age of 20 years with pigmentary retinopathy. Individuals affected may also show other clinical features such as complete heart block, high protein levels in cerebrospinal fluid, cerebellar ataxia, deafness, intellectual delay, and endocrine abnormalities.^45^

On the other hand, mitochondrial encephalomyopathy, lactic acidosis, and stroke-like episodes present in children or young adults as recurrent episodes of encephalopathy, myopathy, headache, and focal neurologic deficits that slowly progress.^41^

**Figure 3:**
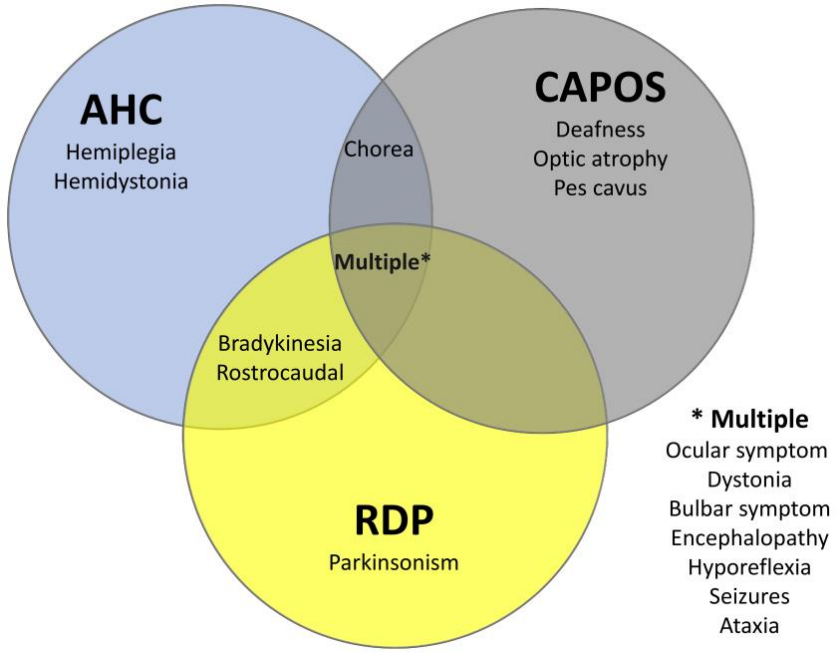
Overlapping of AHC, CAPOS, and RDP. AHC, alternating hemiplegia of childhood; CAPOS, cerebellar ataxia, areflexia, pes cavus, optic atrophy, and sensorineural hearing loss; RDP, rapid-onset dystonia-parkinsonism.

Clinical differentiation of AHC should include other disorders of the group of *ATP1A3*-related disorders.^46^ The disorders that most commonly overlap in clinical manifestations with AHC are RPD and CAPOS syndrome (Figure 3) (Table 3).^47^ But other conditions should also be considered due to similarities in clinical presentation, such as early infantile epileptic encephalopathy (EIEE), relapsing encephalopathy with cerebellar ataxia, and childhood rapid-onset ataxia.^48^,^49^

**Table 3:** Clinical Characteristics of Alternating Hemiplegia of Childhood (AHC), Cerebellar Ataxia, Areflexia, Pes Cavus, Optic Atrophy, and Sensorineural Hearing Loss (CAPOS) Syndrome, and Rapid-onset Dystonia-Parkinsonism (RDP).

Clinical Characteristic	AHC	CAPOS Syndrome	RDP
Age of onset	Before 18 months	Infancy–childhood (between 1 and 5 years of age)	After 18 months (often second to third decade)
Triggers	Excitement, extreme heat or cold, fever, food, lighting changes, physical exertion, stress, water exposure	Fever	Fever, running, alcohol binges, mild traumatic brain injury, overheating, emotional stress, infections, sleep deprivations, and childbirth
Onset	Acute	Acute–subacute ataxia	Abrupt onset over a few minutes to 30 days
Distribution	Hemiplegia/quadriplegia, rostrocaudal gradient, bulbar symptoms	Symmetric, ascending with or without bulbar symptoms	Asymmetric, rostrocaudal gradient, bulbar symptoms
Motor features	Dystonia, choreoathetosis	Ataxia	Dystonia, parkinsonism
Ocular involvement	Paroxysmal abnormal eye movement	Paroxysmal ophthalmoplegia	Paroxysmal abnormal eye movement
Peripheral nervous system	No	Areflexia, optic atrophy, sensorineural hearing loss	No
Pes cavus	No	Yes	No
Seizures	Yes	Inconsistent	Inconsistent
Other manifestations	Cognitive impairment, autonomic phenomena	None	None
Early symptoms	Paroxysmal ocular manifestations, seizures, developmental delay	Fever-induced transient encephalopathy	Vague symptoms of dystonia in distal limbs
Course	Polyphasic (relapsing-remitting)	Relapsing course of ataxia-encephalopathy (one to three episodes) with slow progression of other features	Rarely “secondary” exacerbations (2–3 episodes occurring 1–9 years after the initial onset)
Atypical manifestation	Benign familial nocturnal AHC; mild AHC; dystonia-predominant AHC; familial dominant pedigree; late-onset AHC; AHC without quadriparesis	Urinary urgency; cardiac arrhythmia; left ventricular arrhythmia; scoliosis; cognitive dysfunction; autistic traits; bradykinesia; myoclonus; chorea; tremor; oral dyskinesias; dystonia	Prominent lower limb dystonia; late onset (>50 years of age); gradual onset; pure dystonia; writer’s cramp; pyramidal signs; myoclonus; ataxia; chorea; hyporeflexia
Genotype-phenotype	S137Y, S137F, Q140L, I274N, E277K, V322D, C333F, T335P, G358C, L371P, G755A, G755S, G755C, L757P, T771N, S772R, N773S, N773I, D801E, D801N, T804I, D805E, M806R, I810F, I810S, S811P, E815K, 2542+1G.A (splice site), 919Vdel, D923N, D923Y, C927Y, C927F, C927W, G947R (2839G.A and 2839G.C), A955D, D992Y, 1013Ydup	E818K	I274T, E277K, 327Ldel, T370N, W382R, L417P, T613M, S684F, R756H, I758S, F780L, D810Y, D923N

Benign familial nocturnal alternating hemiplegia of childhood (BNAHC) is considered a probably migraine-related disorder presenting with recurrent attacks of hemiplegia arising from sleep and observed in children in the absence of neurological or cognitive impairment (Table 4).^1^,^6^,^7^,^50^–^53^ Maas et al. reported on two cases, and a further 12 cases from the literature, stating that the age of onset of BNAHC ranged from 4 months to 3 years.^54^ Episodes of hemiplegia occurred during nocturnal or daytime sleep and were associated with inconsolable crying.^42^ Familial or sporadic hemiplegic headache is a subtype of migraine with aura. Individuals with this disorder experience weakness on one side of the body just before or during migraine headaches, and the degree of weakness can vary from mild to severe. Reports of weakness are also associated with other types of aura and visual symptoms, which may affect only a part of the body and, more rarely, the entire body.^55^ The broader use of genetic technology has enabled the differentiation of the AHC diagnosis from similar disorders and its clinical spectrum to be extended.

**Table 4:** Clinical Features of Atypical Cases of Alternating Hemiplegia of Childhood (AHC).

Condition	Clinical Features to Differentiate from Classic AHC	References
AHC without quadriparesis	No report of bilateral hemiplegia or quadriplegia. Interestingly, the patient will fulfill all other clinical criteria for AHC.	Mikati et al. (2000)^1^
Autosomal dominant AHC	Fulfillment of all clinical criteria for AHC. A familial inheritance pattern of autosomal dominant transmission is observed.	Mikati et al. (2000)^1^ Swoboda et al. (2004)^50^
Benign familial nocturnal AHC	It is a familial disease that will be present in first-degree relatives. Hemiplegia most commonly occurs after awakening. The patient will present normal development without fixed neurological deficits.	Andermann et al. (1994)^51^ Villéga et al. (2011)^53^
Dystonia-predominant AHC	The patient presents with isolated dystonia. Hemiplegia only occurs later in life.	Mikati et al. (2000)^1^ Kansagra et al. (2013)^6^
Late-onset AHC	The patient can develop paroxysmal attacks, but neurological findings or delayed developmental milestones are only observed after 18 months of age.	Mikati et al. (2000)^1^ Saito et al. (1998)^52^
Mild AHC	Infrequent episodes of hemiplegia, which may be prolonged.	Mikati et al. (2000)^1^ Bourgeois et al. (1993)^7^

## EPIDEMIOLOGY

The prevalence of AHC is 1:1,000,000 in children under the age of 16 years. Still, this number could be underestimated due to the variability in clinical presentation and the lack of genetic analysis in the preceding epidemiologic data.^56^

One of the largest AHC studies was conducted by Sweney et al., who reported on 103 individuals (56 females and 47 males).^5^ According to these authors, the most frequent and early symptoms observed in the first 3 months of life were paroxysmal eye movements in 83% and hemiplegic episodes by 6 months in 56% of patients.^5^ Dystonic symptoms preceded hemiplegic episodes in 35/86 (41%) cases, and both dystonic and plegic episodes had a co-occurrence onset in 30/86 (35%).^5^

Aicardi et al. summarized the results of 75 AHC subjects previously described in the literature, indicating clinical features and frequency: hemiplegia involving either side (100%); episodes of double hemiplegia/quadriplegia (93%); tonic/dystonic attacks (96%); oculomotor abnormalities (nystagmus/gaze deviation/strabismus) (97%); the remission of symptoms with sleep (94%); developmental delay or intellectual disability (98%); and neurological deficits/choreoathetosis/dystonia (90%).^10^

Pavone et al. revealed intrafamilial clinical variability in the clinical course and long-term outcomes of AHC in twin sisters. In these twins, clinical manifestations of AHC started in the early days of life with episodes of bath-induced abnormal ocular movements that persisted from the first months to 2 years of age.^57^

## MANAGEMENT

### Managing Acute Attacks

Therapeutic management is complex, and every aspect of AHC should be considered. Although no specific disease-modifying agents have been approved for this condition, flunarizine may help in symptomatic management. Prophylactic treatment and acute management of attacks may be addressed in AHC therapy.^46^ While several drugs have been suggested, it has been shown that calcium channel blockers have the highest efficacy. Flunarizine is the most often used medication, with a dosage range of 5–20 mg/day, and 10 mg being the most commonly used. Acute therapy should aim to eliminate or reduce the recognized triggers and encourage sleep hygiene measures. Flunarizine was shown to lessen the duration and intensity of symptoms in some cases; most commonly, it was shown to reduce the duration and severity of hemiplegic bouts.^14^,^58^

Benzodiazepines and other sleep inductors have already been reported to be effective in managing acute attacks of AHC. One of the hypotheses is that the mechanism of facilitating sleep induction helps decrease the number of attacks since paroxysmal attacks frequently disappear during sleep, although attacks may resume soon after awakening.^59^

### Preventing Episodes by Long-term Medication and Avoiding Triggers

**Figure 4:**
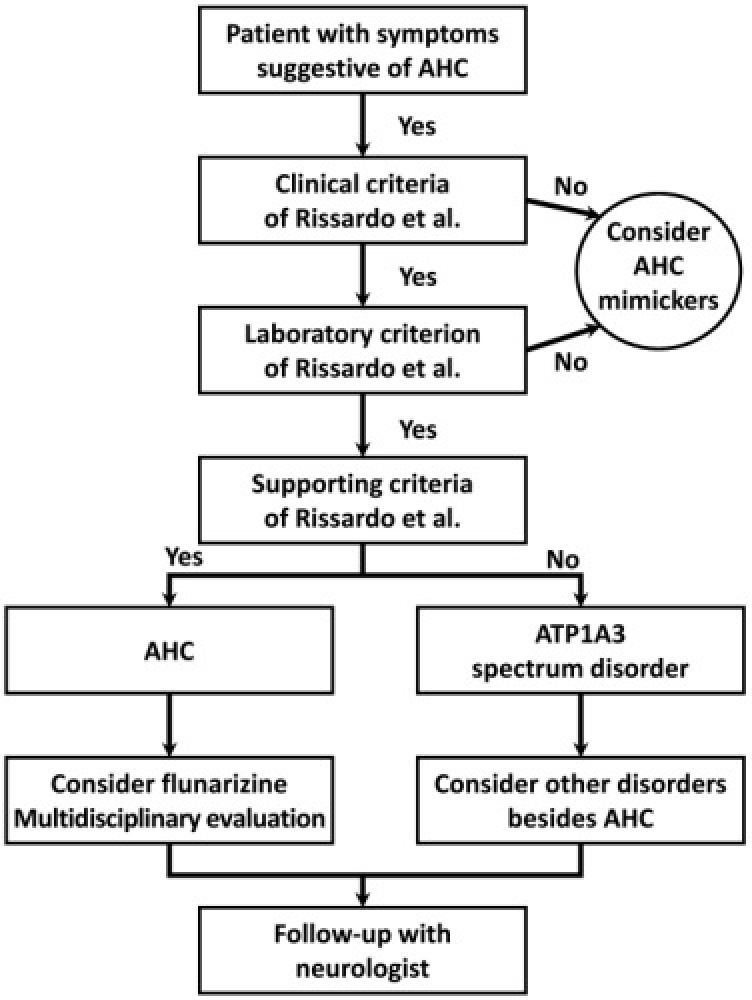
Management According to the Diagnosis Criteria Proposed by Rissardo et al.

Bourgeois et al. treated 17 children with AHC with flunarizine.^60^ They found that only one child experienced a significant decrease in the frequency of hemiplegic attacks (by more than 50%) and that nine of the children experienced a significant reduction in the severity and duration of the hemiplegic attacks (from several days to a few hours on average).^60^ In a large Japanese cohort with 23 AHC cases, more than 75% of the individuals showed a reduction in the frequency and severity of the attacks after flunarizine therapy.^61^ Mikaki et al. showed similar benefits with flunarizine, with which a clinically significant decrease in the frequency and/or severity of AHC attacks was achieved in 21 out of 44 patients. Flunarizine completely prevented hemiplegic attacks in 1 patient (4%), only slightly improved attacks in 2 patients (7%), and had no effect in 4 patients.^1^ Sweney et al. observed a reduction in the frequency of dystonic and hemiplegic episodes in 60% of the patients using flunarizine and in 38% with long-term benzodiazepine therapy.^5^ Notably, several other therapies have already been reported in the literature for managing AHC, but their effectiveness has not been fully established (Figure 4). These include topiramate, ketogenic diet, triheptanoin steroid, oral adenosine-5′-triphosphate supplementation, amantadine, memantine, aripiprazole, coenzyme Q, acetazolamide, dextromethorphan, and vagus nerve stimulation.^59^

Neville and Ninan recommend using flunarizine at the beginning of each episode, treating the epileptic seizure, trying to avoid trigger events, and advising adequate sleep.^56^ Flunarizine is the most frequently prescribed medication for the management of paroxysmal plegic attacks in AHC. Pisciotta et al. observed that flunarizine showed a statistically significant effect in lowering the frequency and duration of paroxysmal attacks in 50% of patients and their intensity in 32%, with considerably increased efficacy related to younger individuals.^62^

Jiang et al. documented the effectiveness of topiramate in four patients.^63^ The medication markedly reduced the frequency and duration of hemiplegic episodes in every patient. They also noticed an increased effect in individuals with seizures, migraines, involuntary movements, and autonomic symptoms.^63^ Therefore, topiramate as a long-term therapeutical choice should be considered for these disorders, when associated with AHC.

Van Hillegondsberg and Michaelis reported using verapamil 6.6 mg/kg/day in three doses in 5-year-old pediatric patients.^64^ They observed a reduction in the frequency, intensity, and duration of episodes, with a 6-month marked abatement of severe debilitating plegic episodes in a significant number of individuals.^64^

Two AHC patients were treated by Samanta and Ramakrishnaiah using intravenous immunoglobulin (IVIG) infusion.^65^ A 2-year-old boy receiving IVIG infusion for 10 months was able to be seizure-free for 2 years after starting treatment, but unfortunately hemiplegic episodes recurred. An 8-year-old girl received periodic IVIG infusion for 4 years, which resulted in remission of paroxysmal events during the first 16 months of treatment.^65^

### Utilizing Sleep as a Management Strategy

Several studies have identified triggers in around half of the patients with AHC, such as exposure to cold, stress, fatigue, bathing, temperature fluctuations, and respiratory infections.^66^ However, excessive avoidance of stressful factors might significantly compromise the quality of life and increase the incidence of psychiatric conditions such as anxiety, depression, and compulsive behaviors. Handling acute episodes is challenging, and developing coping strategies is essential, which can be facilitated by cognitive behavioral therapy. Early sleep induction is recommended due to its consistent alleviation of motor symptoms.^67^ Methods include oral midazolam or melatonin administration. Though these are not officially approved for this purpose, they can be used on a case-by-case basis.^59^ Also, pain relief using paracetamol or ibuprofen might mitigate the distress during attacks.^56^

### Addressing Epilepsy

Epilepsy affects about half of the patients with AHC, often manifesting distinctly from AHC attacks.^68^ In this context, there are reports of status epilepticus in individuals with AHC. However, there is no universally accepted drug for preventing AHC episodes, and epilepsy is usually suboptimally treated.^69^ Motor symptoms persist between attacks and require assessment and management that takes into account mobility, dyspraxia, dystonia, chorea, tremors, weakness, and spasticity.^70^ Surgical intervention remains uncommon due to uncertainties about its effectiveness during acute episodes.

Vagus nerve stimulation was studied in individuals with AHC and epilepsy. Helseth et al. revealed a more than 80% reduction in seizure frequency and 60% reduction in hemiplegic and dystonic episodes. The nerve stimulation parameters were not provided, but the adverse events were mild, with a low increase in the apnea-hypopnea index.^71^

Topiramate was the most commonly reported antiseizure medication with AHC. A common pathophysiology between seizures and AHC has been hypothesized due to the therapeutic benefits of topiramate in both of these conditions. Other antiseizure medications used were ethosuximide, felbamate, phenobarbital, phenytoin, and valproic acid. Interestingly, no antiseizure effect in the ATP was observed in different studies.^68^

### Multidisciplinary Approach

The AHC disorder can be differentiated from other movement disorders in several aspects, mainly due to the multifactorial involvement of several aspects of daily life. Therefore, programs with patients with AHC would benefit from multidisciplinary teams because they can optimize symptomatic and preventive management of this syndrome (Table 5).^12^,^19^,^59^,^68^

**Table 5:** Management of Disorders Commonly Found in Patients with Alternating Hemiplegia of Childhood.

Condition	Management	Reference
Epilepsy	Antiseizure medications, vagus nerve stimulation, and ketogenic diet	Uchitel et al. (2019)^68^
Attention-deficit/hyperactivity disorder	The management of this disorder in patients with alternating hemiplegia of childhood should be similar to that of any other patient without alternating hemiplegia of childhood	Jasien et al. (2019)^19^
Aggressive behavior	Anecdotal reports favor aripiprazole rather than risperidone or haloperidol	Samanta (2020)^59^
Cognitive and learning impairments	Occupational, physical, and speech therapies	Masoud et al. (2017)^12^
Movement disorders (chorea and dystonia)	Dopaminergic agents, tetrabenazine, benzodiazepines, and antiseizure medications	Samanta (2020)^59^
Migraine	The most commonly used agents are topiramate, coenzyme Q, and valproate; other agents already reported are amitriptyline and verapamil	Samanta (2020)^59^

One example of the significance of multidisciplinary evaluation is the nutritional deficiencies in this group of individuals. Most AHC-affected children tend to be small and underweight due to reduced calorie intake during episodes.^43^ Nutritional consultation by a dietitian with observation of daily calorie intake is beneficial for the continuous development of the patient. Also, if necessary, gastrostomy should be considered if conservative management is not effective, mainly in those individuals with significant oropharyngeal dysfunction due to a high risk of aspiration pneumonia.

A formal neuropsychological assessment is another critical aspect that is sometimes neglected in individuals with rare disorders. Board-certified neuropsychologists are recommended to perform the tests in every patient with AHC at the diagnosis, which should be repeated periodically. A significant number of patients with AHC will develop attention-deficit/hyperactivity disorder and mood disorder that sometimes can be difficult to distinguish and can be misdiagnosed as cognitive impairment.

### Medications Used in AHC Treatment

Several agents have already been attempted to manage AHC, and there are no disease-modifying therapies for this syndrome. However, the discovery of the *ATP1A3* mutation in AHC in the last decade may significantly alter the treatment of this disorder in the subsequent years. Prophylactic agents are summarized in Table 6.^5^,^33^,^58^,^62^,^63^,^72^–^85^

**Table 6:** Medications Used in the Management of Alternating Hemiplegia of Childhood.

Drug	Dose	Mechanism	Study Type	Reference
Acetazolamide	187.5 to 500 mg/day	Carbonic anhydrase inhibitor	Case report	Camfield and Andermann (2006)^72^
Amantadine	5 mg/kg/day and 120 mg/day	Non-competitive NMDA receptor antagonist	Case report	Sone et al. (2000)^73^
Aripiprazole	2 to 12.5 mg/day	Dopamine D2 receptor partial agonism	Case report	Dundar et al. (2019)^74^ Haffejee and Santosh (2009)^75^
Benzodiazepine	Variable	GABA-positive allosteric modulator	Case series and cohort studies	Pisciotta et al. (2017)^62^
Betamethasone	NA	Positive modulator of Na^+^-K^+^-ATPase	Case report	Wong and Kwong (2015)^76^
Coenzyme Q	300 mg/day	Antioxidant and part of the mitochondrial respiratory chain	Case report	Bhatt et al. (2007)^77^
Flunarizine	5 mg every other day to 20 mg/day	Selective calcium channel blocker	Case series and cohort studies	Casaer (1987)^58^
Ketogenic diet	Carbohydrate restriction, 10 to 30 mg/day	Increases activation of adenosine triphosphate-sensitive potassium channels, reduces neuronal excitability and firing, and stabilizes synaptic function	Case series	Schirinzi et al. (2018)^78^ Roubergue et al. (2015)^79^ Ulate-Campos et al. (2014)^80^ Vila-Pueyo et al. (2014)^33^
Memantine	10 mg twice daily	Non-competitive NMDA receptor antagonist	Case report	Korinthenberg (1996)^81^
Oral ATP	2 to 25 mg/kg/day	Increases muscle strength via vasodilation and increased blood flow	Case report	Ju et al. (2016)^82^
Topiramate	1 to 20 mg/kg/day; 50 to 150 mg/day	Blocks voltage-dependent sodium channels	Case series and cohort studies	Pisciotta et al. (2017)^62^ Chi et al. (2012)^83^ Sweney et al. (2009)^5^ Di Rosa et al. (2006)^84^ Jiang et al. (2006)^63^
Triheptanoin	30% of daily calorie intake	Fatty acid metabolism and anaplerosis	Double-blind placebo-controlled trial	Hainque et al. (2017)^85^

GABA, gamma-aminobutyric acid; NA, not applicable/not reported; NMDA, N-methyl-D-aspartate.

## PROGNOSIS

Alternating hemiplegia of childhood is a multifaceted and varied disorder that goes beyond hemiplegic episodes, encompassing a range of clinical dysfunctions that notably involve the autonomic nervous system and musculoskeletal system, often leading to seizures and cognitive impairments. Patients with AHC exhibit a spectrum of symptoms, varying from mild to severe manifestations, alongside associated disorders.

The multi-level dysfunctions can significantly impact the long-term well-being of children affected by AHC. While hemiplegic episodes tend to decrease with age, a recent study by Cordani et al. reported epileptic seizures in 62% of the patients with AHC.^1^,^8^,^29^ Also, the authors observed that seizures can potentially play a detrimental role in the pathophysiological progression, particularly in individuals with drug-resistant epilepsy.^29^ Although there is no evidence suggesting AHC affects life expectancy, patients may experience complications related to cognitive impairment and oropharyngeal dysfunction, leading to life-threatening aspiration. Pavone et al. revealed that patients with persistent neurological findings compared to paroxysmal episodes, in which migraine, fine and gross motor dysfunction, walking disturbances, speech problems, and cognitive impairments are found, will more commonly have these features throughout their lives.^86^

In sporadic AHC cases, the clinical course tends to be more severe than in familial cases.^7^ The prognosis significantly depends on the age of onset, especially when hemiplegic spells begin early.^87^ Children displaying neonatal-onset symptoms experience severe developmental impacts, mainly when recurrent convulsive status epilepticus occurs, leading to psychomotor deterioration.^43^ While some children affected by motor dysfunctions require wheelchair assistance, others achieve independence in adulthood.^20^ As individuals grow older, hemiplegic episodes, abnormal ocular movements, and hypotonia become less frequent and less severe. The natural progression of AHC is often characterized by unpredictable relapses and remissions, not consistent until adulthood, suggesting the potential existence of different AHC subtypes.^2^

## AHC AND ATP1A3-RELATED DISORDERS

**Figure 5:**
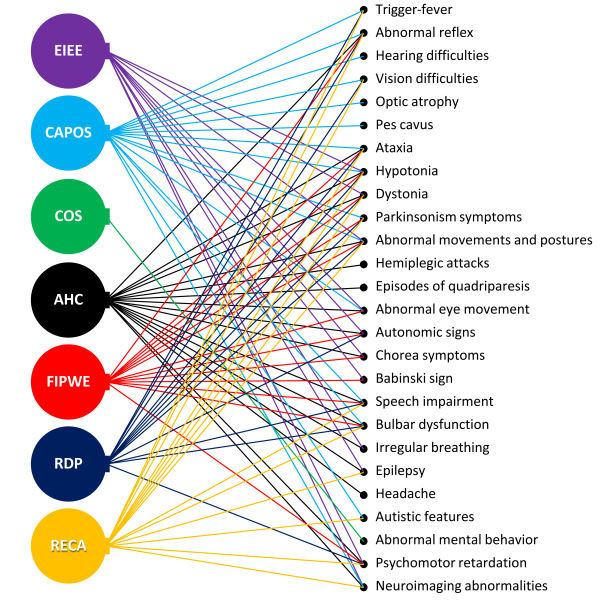
*ATP1A3* Spectrum Disorders. AHC, alternating hemiplegia of childhood; CAPOS, cerebellar ataxia, areflexia, pes cavus, optic atrophy, and sensorineural hearing loss syndrome; COS, childhood-onset schizophrenia; EIEE, early infantile epileptic encephalopathy; FIPWE, fever-induced paroxysmal weakness and encephalopathy; RDP, rapid-onset dystonia-parkinsonism; RECA, relapsing encephalopathy with cerebellar ataxia. Diagram based on the study of Li et al. (2022).^88^

Mutations within ATP1A3 are prevalent in AHC cases, accounting for approximately 78%, as noted in one study,^36^ and rising to 92% according to a recent Italian investigation.^29^ Besides AHC, these mutations have associations with other clinical conditions, forming a spectrum known as *ATP1A3*-related disorders (Figure 5).^88^ This spectrum encompasses distinct entities like AHC, RPD, and CAPOS syndromes.^35^,^47^ Additionally, EIEE, relapsing encephalopathy with cerebellar ataxia, and childhood rapid-onset ataxia have similar clinical manifestations and should be considered part of this spectrum. There are also reports of intermediate phenotypes or individuals showing atypical features not entirely fitting the classical signs of the disorders mentioned above.^39^,^49^,^89^

### Fever-induced Paroxysmal Weakness and Encephalopathy

Patients with the *ATP1A3* R756H mutation experience childhood onset with sporadic fever-induced episodes causing encephalopathy and weakness. Long-term outcomes vary, ranging from mild motor issues to dysphagia. Some exhibit symptoms like dysarthria, cognitive problems, motor apraxia, and gait difficulties.^90^

### Slowly Progressive Cerebellar Ataxia Without Paroxysms

Two patients carrying different *ATP1A3* variants showed slow, continuous cerebellar ataxia without episodic symptoms. Magnetic resonance imaging (MRI) scans revealed mild cerebellar cortical atrophy in both cases.^91^

### Childhood-onset Schizophrenia/Autistic Spectrum Disorder

Cases of childhood-onset schizophrenia (linked to *ATP1A3* mutations) exhibit symptoms such as selective mutism, severe behavioral disturbances, aggression, auditory hallucinations, and mild motor delay. Some patients also have autistic spectrum disorder alongside these clinical symptoms.^92^,^93^

### Paroxysmal Dyskinesia

Cases of hyperkinetic movements triggered by various factors involving painful postures lasting minutes to hours were reported. Genetic analysis revealed *ATP1A3* mutations linked to these symptoms. Another family presented exercise-induced dystonia without plegic attacks.^24^,^94^

### Cerebral Palsy/Spastic Paraparesis

Several cases with *ATP1A3* mutations showcased symptoms like spastic diplegia, developmental delay, epilepsy, and episodic neurological deterioration. One case exhibited static encephalopathy, microcephaly, and dystonia.^95^

### Dystonia, Dysmorphism, Encephalopathy, MRI Abnormalities, and No Hemiplegia

Patients with *ATP1A3* variants presented with dystonia, seizures, and facial dysmorphism; cerebellar abnormalities were detected through MRI scans. Symptoms varied among patients, including hypotonia, tremors, nystagmus, and epileptic seizures.^96^ However, no hemiplegia was reported in this subgroup of individuals.

### Congenital Hydrocephalus and Other Brain Abnormalities

Reports in the literature highlighted cases of severe congenital hydrocephalus, Chiari malformation, schizencephaly, and corpus callosum dysgenesis linked to *ATP1A3* mutations. Additionally, patients with heterozygous *ATP1A2*/*ATP1A3* mutations showed developmental and epileptic encephalopathy, often associated with cortical developmental malformations leading to early lethality in some cases.^97^,^98^

## ANIMAL STUDIES

Animal models are valuable tools for studying *ATP1A3* disorders, especially due to the high degree of similarity between the genomics of humans and animal models (Table 7).^4^,^99^–^104^ Specifically, the amino acid sequence similarity between the mouse and human Na^+^/K^+^-ATPase alpha-3 subunits is approximately 99%.^105^ The first animal model for *ATP1A3*-related disorders was developed by Moseley et al.^106^ This mouse model was based on a point mutation in *ATP1A3* intron 4 (targeted knock-in), which was subsequently replicated by Kirshenbaum et al. and DeAndrade et al.^99^,^107^ The animal model *ATP1A3*tm/1Ling/^+^ has increased ambulation in the open field, increased methamphetamine response, and increased hidden latency in the water maze. However, it does not develop spontaneous seizures.^108^

**Table 7:** Summary of Pre-clinical Mouse Models of *ATP1A3*-related Disorders.

Model	Mouse Genome Informatics	Phenotype	Spontaneous Seizures	Reference
*ATP1A3*^tm/1Ling/+^ (Alpha3^+/KOI4^)	3696954	Motor hyperactivity, mania-like behavior, sleep cycle abnormalities	No	Kirshenbaum et al. (2011)^99^
*ATP1A3* ^tm2Kwk^	5572809	Motor hyperactivity, no gross morphological defects or apparent histological brain anomalies, no development of dystonia spontaneously	No	Ikeda et al. (2013)^100^
*ATP1A3*^D801Y^ (alpha3^D801Y^; *ATP1A3*^tm1Klh^)	6163502	Hyperactivity, increased sensitivity to chemically induced epileptic seizures, and cognitive deficits	No	Holm and Lykke-Hartmann (2016)^101^
Myshkin (*ATP1A3*^Myk^)	4356167	Motor hyperactivity, spontaneous and induced by vestibular stress, hippocampal hyperexcitation, memory and learning reduction	Yes	Clapcote et al. (2009)^102^ Kirshenbaum et al. (2011)^99^
Mashlool (Mashl; *ATP1A3*^tm1Ute^)	6162645	Small size, hyperactivity, ataxia and tremor, hind-limb clasping (tail suspension), reversible hemiplegia, quadriplegia, and dystonia, avoidant and anxiety-like behaviors	Yes	Hunanyan et al. (2015)^4^
Matoub (Matb; *ATP1A3*^E815K^; *ATP1A3*^tm1Mika^)	6197036	Small size, hemiplegia/dystonia/seizures spontaneously and upon stimulation, abnormal episodic memory, abnormal motor performance and coordination, less activity and freezing; high mortality	Yes	Helseth et al. (2018)^103^
ROSA26-*ATP1A3*^D591V^ [Gt(ROSA) 26Sor^tm1(CAG-^*^ATP1A3^*^-D591V,-EGFP)^]	NA	Scarce description in the literature regarding the phenotype	No	Zhou et al. (2020)^104^

NA, not available/not applicable.

Other models of ATP-related disorders present in the literature are drosophilas and zebrafish. Interestingly, all the flies with a mutation in human Na^+^/K^+^-ATPase alpha-3 subunits (Ala588Thr, Gly528Ser, Gly744Ser, Pro262Leu, Ser201Leu, Ser348Thr) showed mechanical stress-induced paralysis. However, only three had temperature-sensitive paralysis (Gly744Ser, Asp981Asn, and Glu982Lys).^105^ Zebrafish models are more challenging to analyze because their *ATP1A3* comprises two orthologues, *ATP1A3*a and *ATP1A3*b. Also, it is suggested that both α3 paralogues are needed for embryonic motility.^109^

## FUTURE STUDIES

Diagnostic criteria with specific tests are urgently needed to help differentiate AHC from other *ATP1A3*-related disorders. The present diagnostic criteria format based only on the clinical criteria leads to significant overlap among the different diseases associated with the *ATP1A3* mutations, as shown in Figure 5.

Another area that should be studied is the management of AHC. There are only retrospective studies with flunarizine, and isolated case reports with some medications. There is an urgent need for medications that can change the course of this condition, decreasing the number of paroxysmal attacks and promoting a better quality of life.

## CONCLUSION

Alternating hemiplegia of childhood is a complex and diverse disorder characterized primarily by hemiplegic episodes, varying widely in frequency and intensity. These episodes commonly coincide with epilepsy, developmental delay, movement disorders, and autonomic nervous system dysfunction. Most AHC cases are associated with *ATP1A3*-related disorders, encompassing RDP, CAPOS syndrome, EIEE, childhood rapid-onset ataxia, and relapsing encephalopathy with cerebellar ataxia.

## Abbreviations

AHC: alternating hemiplegia of childhood
CAPOS: cerebellar ataxia, areflexia, pes cavus, optic atrophy, and sensorineural hearing loss
EIEE: early infantile epileptic encephalopathy
IVIG: intravenous immunoglobulin
MRI: magnetic resonance imaging
RDP: rapid-onset dystonia-parkinsonism.
